# State Regulatory Response to COVID-19 Limited Assisted Living Resident Caregiver Networks

**DOI:** 10.1093/geroni/igab046.218

**Published:** 2021-12-17

**Authors:** Lindsey Smith, Bethany Linscott Lowe, Sarah Dys, Kali Thomas, Sheryl Zimmerman, Paula Carder

**Affiliations:** 1 OHSU-PSU School of Public Health, Portland, Oregon, United States; 2 Oregon Health & Science University - Portland State University School of Public Health, Portland, Oregon, United States; 3 Portland State University, Portland, Oregon, United States; 4 Brown University, Brown University/Providence, Rhode Island, United States; 5 Cecil G. Sheps Center for Health Services Research, Chapel Hill, North Carolina, United States

## Abstract

This paper describes a qualitative content analysis of assisted living emergency rules, revised regulations, and executive orders responding to the COVID-19 pandemic. Using key search terms, we identified 36 states that enacted policies between February and October 2020. The following themes occurred most frequently: testing, infection control, access restrictions, suspension of requirements, and reporting. The convoys of care model recognizes internal, external, formal, and informal caregivers as essential members of an AL resident’s care network. We found that non-staff care providers, including external formal caregivers (e.g. home health and hospice) and informal caregivers (e.g. family), were most often addressed in policies limiting access. Informal caregivers were the least often specifically addressed in these policies. Given the importance of these network members in the AL context, these policies have implications for the wellbeing of the resident and care network.

